# Don't Let Perfect Be the Enemy of Good: A Proof of Concept for a Custom National Data Repository of Quality Measures for Free and Charitable Clinics

**DOI:** 10.1089/heq.2022.0078

**Published:** 2022-09-15

**Authors:** Julie S. Darnell, Michael Perry, Nicole Lamoureux, Edith Lee

**Affiliations:** ^1^ Department of Public Health Sciences, Parkinson School of Health Sciences and Public Health, Loyola University Chicago, Chicago, Illinois, USA.; ^2^ Department of Mathematics and Statistics, College of Arts and Sciences, Loyola University Chicago, Chicago, IL, USA.; ^3^ National Association of Free and Charitable Clinics, Alexandria, Virginia, USA.; ^4^ Americares, Stamford, Connecticut, USA.

**Keywords:** free clinics, uninsured, quality improvement, quality indicators, healthcare disparities, health equity

## Abstract

**Purpose:**

Free and charitable clinics (FCCs), nonprofits that utilize volunteer licensed health care professionals to provide health services at no cost or a small fee to low-income uninsured patients who are disproportionately from underrepresented communities, have been part of the safety net for over a century. Approximately 1400 known FCCs serve two million patients annually. Despite their longevity and sizable number, evidence regarding the quality of care in FCCs is lacking. We report new evidence generated by a national initiative, the Roadmap to Health Equity. Established in 2017, this consortium is co-led by two national organizations serving FCCs and an academic institution. It has involved more than 150 FCC stakeholders with the shared goal of improving the quality of care and reducing inequities. The centerpiece is a custom national data repository of 15 validated clinical quality measures and patient-level characteristics.

**Methods:**

Fifty FCCs pilot tested the data repository. Clinics submitted patient-level data on two blood pressure (BP) measures and at least one additional measure. Descriptive statistics were stratified by sex, race, ethnicity, and language.

**Results:**

In 2021, 33 pilot FCCs from 21 states reported data across 13 of the 15 clinical measures, representing 34,359 unique patients. For example, on average, 60% of patients had controlled BP, but Black patients had lower rates of BP control than Hispanic and White patients (55.9% vs. 62.1% and 63.0%, respectively).

**Conclusion:**

Our findings demonstrate a proof of concept. By standardizing quality measures alongside patient characteristics, clinics can become aware of racial/ethnic inequalities in health outcomes. This information can motivate clinics to investigate the causes and implement solutions. In an environment where outcome data from FCCs are scarce, the new national data repository lays the foundation for routine stratified reporting of a range of quality outcomes for an important safety net for the uninsured.

## Introduction

Free and charitable clinics (“FCCs”), volunteer-based nonprofits that provide a range of health care services at no cost or for a small fee to low-income uninsured patients who are disproportionately members of underrepresented minority groups, have been part of our nation's safety net for over a century.^1^ The ∼1400 known FCCs serve more than two million patients each year.^2^ Despite their longevity and sizeable number of patients, evidence about the quality of care in FCCs is deficient.

The limited literature mostly describes the quality of care at individual clinics, often using small samples.^3–5^ Moreover, these individual accounts overrepresent the experiences at student-run free clinics,^6–12^ which comprise <10% of FCCs and have less capacity—that is, smaller budgets, fewer patients, and fewer visits.^1,13^ In addition, among FCC studies assessing quality, many focus on patient satisfaction,^14–17^ an important but incomplete measure of quality.

Data on the quality of care in FCCs beyond single-clinic narratives are scarce. Statewide-level data exist only in North Carolina where, since 2011, the North Carolina Association of Free and Charitable Clinics (NCAFCC) report on select patient outcomes.^18^ As NCAFCC data represent the largest effort to aggregate standardized quality outcomes, their results best approximate the level of quality of care in FCCs. In their 2020 report based on 65 FCCs, the NCAFCC found that 30.9% of diabetic patients had HbA1c levels above 9%. This is comparable to 34.3% of patients at Health Resources and Services Administration (HRSA) federally funded health centers in North Carolina.^19^ For hypertensive patients, 55.3% had controlled blood pressure (BP) (<140/90)^18^ versus 58.9% at federally qualified health centers (FQHCs).^19^

Limited data on the quality of care among the vast majority of FCCs across the country creates numerous obstacles. For FCCs, the lack of quality data inhibits clinics' abilities to (1) benchmark against their peers, thereby improving care; (2) secure resources from funders that increasingly expect evidence showing outcomes; (3) form partnerships with hospitals and health systems to extend the scope of services; and (4) attract volunteer licensed providers.^20^ For other stakeholders, this lack of data means policymakers, other safety net providers, private philanthropy, the media, and the general population have an incomplete picture of the quality of care. The absence of quality outcome data helps perpetuate myths and misconceptions about FCCs because “free” care is often presumed to be of low quality.^21^

An explanation for the lack of data on the quality of care in FCCs is the lack of a national infrastructure to systematically collect standardized evidence-based data that can indicate the level of quality of care that FCCs provide to their patients (akin to the Uniform Data System [UDS] used by HRSA for health centers; see data.HRSA.gov). To address this shortcoming, a consortium led by two national organizations serving FCCs (Americares, National Association of FCCs) and an academic institution (Loyola University Chicago) joined FCC leaders and state associations representing FCCs in 2017 to create a national initiative called Quality of Care in FCCs: Roadmap to Health Equity: (hereinafter “Roadmap”).

To date, more than 150 FCC stakeholders have worked together to improve quality of care and reduce inequities in FCCs. The centerpiece is a custom national data repository of 15 externally validated clinical quality measures, which are a mechanism to assess processes, experiences, and/or outcomes of patient care, such as BP control, combined with patient-level characteristics, including race, ethnicity, and language. In this study, we report our progress, share early results, and discuss the next steps.

Roadmap was launched in preparation for a National Institute on Minority Health and Health Disparities-sponsored conference. At the 2018 conference, key FCC stakeholders connected to address the lack of national-level quality data for FCCs. There, Roadmap stakeholders reached consensus on a “starter set” containing 15 clinical quality measures, sociodemographic factors, and clinic-level characteristics (Table 1) and agreed to a process for identifying a vendor that would house the quality data. Following the conference, Roadmap participants continued to work through committees aptly named the “How” Committee, which explored how the data would be collected; the “What” Committee, which examined what data ought to be reported into the repository; and the “Why” Committee, which articulated the value proposition.

**Table 1.** Starter Set of Clinical Quality Measures, Sociodemographic Factors, and Clinic Demographic Characteristics with Results by Race, Ethnicity, and Language (*n*=34,359 Patients)

Clinical quality measures					
Clinical quality measure	Source for measure	No. of clinics reporting	No. of patients	Mean %	
				All patients	By race, ethnicity and language^a^
Blood pressure screening and follow-up	CMS 22v8; MIPS 317	33	18,343	Not reported^b^	
Blood pressure control (systolic BP <140 mmHg and diastolic BP <90 mmHg)	CMS 165v8; MIPS 236; NQF 0018	22	2328	60.0	White: 63.0 Black: 55.9 Latinx: 62.1 Other: 64.3 Unknown: 59.7 Potential language barrier: 61.0 No barrier: 58.1
Documentation of current medications in medical record	CMS68v8; NQF 0419e	10	8953		Not reported^b^
Diabetes: hemoglobin A1c testing	NQF 0057	9	842	85.9	All patients are unknown race with potential language barrier
Diabetes: hemoglobin A1c poor control (>9.0%)	CMS 122v8; MIPS 001; NQF 0059	9	1367	34.4	White: 21.1 Black: 0 Latinx: 23.5 Other: 0 Unknown: 36.9 Potential language barrier: 34.7 No barrier: 25.5
Breast cancer screening	CMS 125v8; MIPS 112; NQF 2372	7	1997	61.3	White: 52.9 Black: 66.7 Latinx: 65.6 Other: 50.0 Unknown: 61.8 Potential language barrier: 61.5 No barrier: 57.6
Hypertension: improvement in blood pressure	CMS65v7; MIPS 373	5	313	48.9	White: 0 Black: 50 Latinx: 55.6 Other: 0 Unknown: 49.3 All patients have potential language barrier
Influenza immunization	CMS147v9; MIPS 110; NQF 0041	4	2386	27.1	All patients are unknown race with potential language barrier
Tobacco use: screening and cessation intervention	CMS138v8; MIPS 226; NQF 0038	3	1751	95.0	All patients are unknown race with potential language barrier
BMI screening and follow-up plan	CMS69v8; MIPS 128; NQF 0421	2	2742	90.8	All patients are unknown race with potential language barrier
Screening for depression and follow-up plan	CMS 2v9; MIPS 134; NQF 0418	2	256	95.7	All patients are unknown race with potential language barrier
Cervical cancer screening	CMS 124v8; MIPS 309; NQF 0032	1	1328	75.1	All patients are unknown race with potential language barrier
Colorectal cancer screening	CMS130v8; MIPS 113; NQF 0034	1	1531	55.9	All patients are unknown race with potential language barrier
Unhealthy alcohol use: screening and brief counseling	NQF 2152; MIPS 431	0	Not applicable		
Avoidance of antibiotic treatment in adults with acute bronchitis	NQF 0058	0	Not applicable		

Sociodemographic factors					
Factor	Permissible question	Permissible response categories		Source for measure	
Age	Taken from date of birth in medical record	Year of birth		American Community Survey	
Sex	What is your sex at birth, on your original birth certificate?	Male Female		*HHS Implementation Guidance on Data Collection Standards for Race, Ethnicity, Sex, Primary Language, and Disability Status,*^24^ adding “on your original birth certificate” to differentiate it from gender identity.	
Ethnicity option 1	Are you Hispanic, Latino, or Spanish origin?	Hispanic Not Hispanic		American Community Survey	
Ethnicity option 2	Are you Hispanic, Latino, or Spanish origin?	No, not of Hispanic, Latino or Spanish origin Yes, Mexican, Mexican American, Chicano Yes, Puerto Rican Yes, Cuban Yes, another Hispanic, Latino/a or Spanish origin		American Community Survey	
Race	What is your race? More than 1 category is acceptable.	American Indian or Alaska Native Asian Black or African American Native Hawaiian or Other Pacific Islander White		Office of Management and Budget Guidance	
Language option 1	Do you speak a language other than English at home?	Yes [if Yes, what is the language? Write in] No		*Health Research and Educational Trust Disparities Toolkit* ^25^ *and HHS Implementation Guidance on Data Collection Standards for Race, Ethnicity, Sex, Primary Language, and Disability Status* ^24^	
Language option 2	Would you like an interpreter?	Yes No		*Health Research and Educational Trust Disparities Toolkit* ^25^	
Language option 3	How well do you speak English?	Very well Well Not well Not at all		*Health Research and Educational Trust Disparities Toolkit* ^25^ *and HHS Implementation Guidance on Data Collection Standards for Race, Ethnicity, Sex, Primary Language, and Disability Status* ^24^	
Language option 4	What language do you feel most comfortable speaking with your doctor or nurse?	Spanish Other (write in)		*Health Research and Educational Trust Disparities Toolkit* ^25^	

Clinic characteristics					
Year clinic opened					
Number of delivery sites					
Annual number of unduplicated patients served at clinic					
Annual number of new patients served at clinic					
Annual number of medical visits					
Annual number of dental visits					
Annual number of mental health/behavioral health visits					
Annual number of other visits					
Annual cash operating budget					
Number of full-time employees					
Annual number of volunteers					
Annual number of volunteer hours					
Total days per week or month clinic is open to see patients					
Total monthly hours open to see patients					

^a^ Defined as patients who indicate they need an interpreter, speak a language other than English at home, or speak English less than “Very Well.”

^b^ Not reported due to irregularities in the data.

BMI, body mass index; BP, blood pressure; CMS, Centers for Medicare and Medicaid Services; HHS, U.S. Department of Health and Human Services; MIPS, Merit-Based Incentive Payment System; NQF, National Quality Forum.

**FIG. 1.**
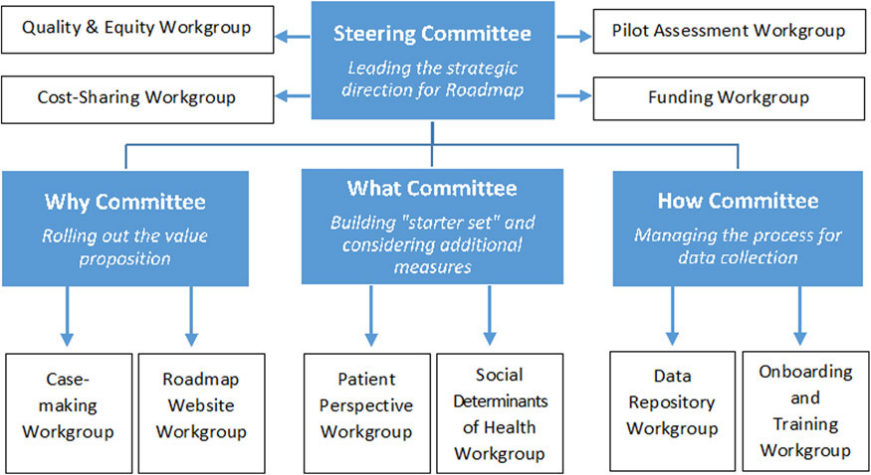
Organizational structure for the roadmap to health equity.

**FIG. 2.**
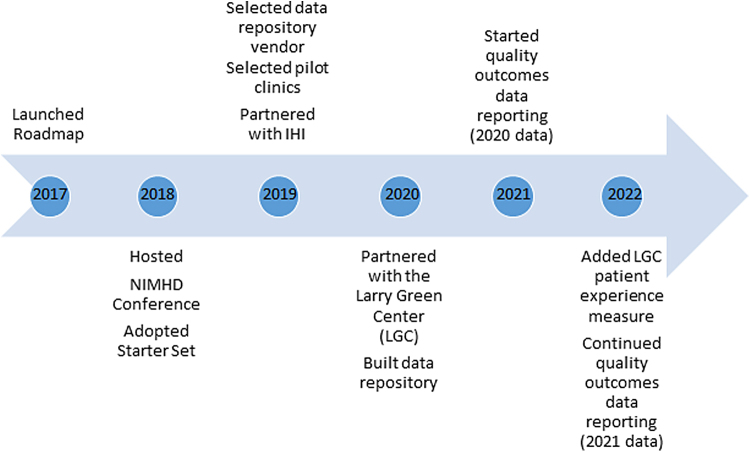
Roadmap to health equity milestones. IHI, the Institute for Healthcare Improvement; LGC, the Larry Green Center; NIMHD, the National Institute on Minority Health and Health Disparities.

A steering committee, comprising nine leaders, the majority of whom are from underrepresented communities, guides the initiative. This original structure (Fig. 1) is intact 5 years later, although the activities subsumed under the committees have changed as the initiative has evolved. Figure 2 highlights the key accomplishments.

## Methods

### Sample

In fall 2019, the Roadmap coleads solicited FCCs through a request for application process to pilot test the data repository created by VisionTree, a company with a cloud-based patient-centered outcomes platform. Of 57 applications received, the coleads selected 50 pilot FCCs. The clinics received $1000 to participate in the pilot phase, which continues through June 2023.

### Materials

In 2021, we asked clinics to submit their 2020 deidentified patient-level data using measure-specific data.csv files on two BP measures and at least one additional clinical quality measure into the new repository (Table 1). The BP measures assess the process and the outcome of patient care. Specifically, the BP screening measure indicates the percentage of adult patients who were screened for high BP and have a recommended follow-up plan documented if BP is elevated. In addition, the BP outcome measure indicates the percentage of hypertensive patients who have controlled BP (i.e., systolic BP is <140 mmHg and diastolic BP is <90 mmHg). A user guide, data dictionary, and training videos provide instructions for submitting the data. Clinics can report the population for a given measure or a random sample of 70 patients using approved methods that mimic the health center UDS methodology.^22^

### Statistical analysis

Using SAS 9.4, we performed descriptive statistics for the clinic characteristics and reported clinical quality outcomes, stratified by sex, race, ethnicity, and language, where possible. We created a single race/ethnicity measure using separate questions on race and ethnicity. We coded patients as having a “potential language barrier” if responses indicated patients needed an interpreter, spoke a language other than English at home, or spoke English less than “Very Well.” The Institutional Review Board of Loyola University Chicago approved the study.

## Results

In 2021, 33 pilot FCCs (66%) from 21 states submitted data across 13 of 15 clinical measures, representing 34,359 unique patients. Table 2 describes the organizational characteristics of the participating FCCs. Pilot clinics submitting data are located in all four regions of the United States, but more than half are from the South. In a logistic regression model, we detected no statistically significant difference by region between pilot clinics that submitted clinical quality data (*n*=33) and pilot clinics that did not submit data (*n*=17). On average, participating clinics have operated for 20 years as of 2020, but the number of years open ranged from 1 year (2019) to 90 years (1930). Half have budgets that are $1 million or greater, but about one-quarter report annual cash operating budgets of <$300,000. More than half of participating clinics are open at least 5 days per week.

**Table 2.** Characteristics of Pilot Clinics (*n*=22)

Characteristic	Mean (SD)/%
Geographic region (*n*=33)	%
Midwest	21.2
Northeast	9.1
South	51.5
West	18.2
Year clinic opened	1992 (21.8) [range 1930–2019]
Number of delivery sites	1.7 (1.6) [range 1–8]
Annual number of unduplicated patients served at clinic	3322 (6269) [range 2–29,793]
Annual number of new patients served at clinic	540 (817) [range 0–3900]
Annual number of medical visits	5802 (9197) [range 12–43,324]
Annual number of dental visits	980 (2026) [range 0–8017]
Annual number of mental health/behavioral health visits	948 (2022) [range 0–9036]
Annual number of other visits	2916 (5984) [range 0–21,158]
Annual cash operating budget	%
0–$149,999	9.2
$150,000–$299,999	13.6
$300,000–$499,999	4.6
$500,000–$749,999	13.6
$750,000–$999,999	9.1
$1,000,000–1,999,999	31.8
$2,000,000+	18.2
Number of full-time employees	15.4 (14.4) [range 0–50]
Annual number of volunteers	213 (236) [range 0–1000]
Number of annual volunteer hours	8360 (11107) [184–40000]
Total monthly hours clinic is open to see patients	117 (70) [range 16–225]
Total days open to see patients	%
1 day per week	4.6
2 days per week	4.6
3–4 days per week	31.8
5 or more days per week	54.6
<1 day per month	4.6

Notes: Geographic location was extracted from clinics' applications to participate in the pilot program. Twenty-two (of 33) clinics submitting clinical quality data completed the clinic demographics form. Percentages may exceed 100% due to rounding.

SD, standard deviation.

More specifically, clinics report being open to serve patients, on an average of 117 h per month. Participating clinics include both volunteer-run enterprises and heavily staffed operations, with a mean of 15 full-time employees and more than 8300 annual volunteer hours. On average, the annual number of total unduplicated patients served is 3322, of which 540 (roughly 15%) are new patients. These patients receive, on average, 5802 medical visits, 980 dental visits, and 948 mental health/behavioral health visits.

Table 1 shows the mean percentage of patients who met each measure, stratified by race/ethnicity and language, if available. For example, among patients with a diagnosis of hypertension, for the BP control performance measure, on average, across 22 reporting clinics, 60.0% of patients had controlled BP, but Black patients had lower rates of BP control than their Hispanic and White counterparts (55.9% vs. 62.1% and 63.0%, respectively). Turning to the diabetes control performance measure, an optional measure, among nine reporting clinics representing 1367 patients with diabetes, 34.4% of patients, on average, had poor control of their diabetes (defined as >9.0% HgA1c). Latinx patients had slightly higher rates of poor diabetes control than White patients (23.5% vs. 21.1%).

Among patients of unknown race, however, 36.9% had poor control of diabetes. When we stratify results by potential language barrier, diabetic patients with a potential language barrier have much higher rates of poor control of diabetes compared with patients without a known language barrier (34.7% vs. 25.5%).

## Discussion

Our findings demonstrate a proof of concept and suggest that the national quality data repository is highly promising in three respects. First, with standardized, evidence-based quality outcomes alongside patient characteristics, clinics can become aware of inequalities in health outcomes based on race, ethnicity, and language. While stratified performance data, by themselves, do not reduce inequities, they can motivate clinics to investigate causes and implement solutions. Second, clinics will be able to compare their individual clinic's performance to other FCCs as well as to related peers, such as health centers. Finally, the new data repository can help clinics meet funders' expectations concerning outcomes, supporting sustainability.

Anecdotal evidence from pilot clinics that are involved in the Roadmap initiative suggests that the COVID-19 pandemic may have hindered clinics' participation (*n*=33 of 50), underscoring the importance of making the data submission process feasible for low-resource settings. Efforts are ongoing to enhance the training materials and simplify reporting. In our preparations for the second wave of data collection, which is currently underway, we have highlighted these improvements in our messaging to pilot clinics, and remain hopeful that we can reengage the pilot clinics that did not submit 2020 data.

Better training materials can also help to prevent data irregularities. For example, we observed that some clinics had misinterpreted the BP screening measure, mistakenly extracting hypertensive patients. In addition, some clinics appeared to report the most recent visit rather than all visits for the documentation of current medications measure. Spotting these issues during the pilot phase offers an opportunity to resolve them before scaling up, ensuring more accurate reporting.

Committed to consensus decision-making, Roadmap has been responsive to FCC leaders' suggestions to improve data accuracy and completeness. For example, clinic leaders have explained that many patients have difficulty answering ethnicity and race with two questions, the federal standard and format we adopted.^23^ In particular, Latinx patients often reportedly do not identify as another race. Consequently, we have observed high levels of “Unknown” race in our 2020 sample, which is a current limitation that constricts our ability to detect racial/ethnic inequalities in the quality of care from the inaugural dataset. As a remedy, starting in the 2021 reporting year, we now give clinics two options to report race and ethnicity: using two questions, or a single question that treats Hispanic origin as a race. We anticipate that this change will result in more complete information about race and ethnicity.

Along with data collection, Roadmap workgroups are bringing resources to clinics to enhance equity and quality improvement efforts. For instance, through a partnership with the Institute for Healthcare Improvement (IHI), four pilot clinics received $30K scholarships, enabling them to receive coaching and peer support through the IHI's Pursuing Equity initiative.

A formal mixed-methods evaluation of pilot clinics is underway to more systematically identify the barriers and facilitators to participating in Roadmap. Feedback from focus groups and surveys will inform our efforts to update our starter set, modify the process for data collection, identify needed resources, refine our messaging about the value of Roadmap, learn about how clinics might use the results to advance their equity and quality improvement activities, determine the optimal pace for scaling up, and decide a cost-sharing strategy to assure long-term sustainability of the national data repository. With initial funding from NIMHD and subsequent external funding from corporate donors as well as from Americares, one of the project coleads, we have the means to add up to 100 pilot clinics over the next year.

Our 4-year pilot phase, begun in late 2019 and continuing through June 2023, enables us to collect quality measures from FCCs across three time periods (2020, 2021, 2022), giving us ample time to identify and address concerns and secure resources for continued growth. This pilot study has several limitations. Similar to the UDS, the data were self-reported. In addition, there are insufficient data to draw definitive conclusions about the quality of care in FCCs, particularly within racial/ethnic groups where the sample sizes can be quite small. Nevertheless, these are the first known national-level quality data and extend the evidence base beyond individual clinics and North Carolina.

## Conclusion

In an environment where outcome data from FCCs are scarce and, if available, often reported in the aggregate, the new national data repository lays the foundation for routine stratified reporting of a range of quality outcomes for an important safety net for the uninsured. This foundation is imperfect, but improved thanks to pilot clinics, and could ultimately help drive increased evidence-based decision-making and better outcomes in FCCs.

## Abbreviations Used

BMI: body mass index
BP: blood pressure
CMS: the Centers for Medicare and Medicaid Services
COVID-19: coronavirus disease 2019
FCCs: free and charitable clinics
FQHCs: federally qualified health centers
HbA1c: hemoglobin A1c
HHS: U.S. Department of Health and Human Services
HRSA: Health Resources and Services Administration
IHI: the Institute for Healthcare Improvement
LGC: the Larry Green Center
MIPS: Merit-Based Incentive Payment System
NACFCC: the North Carolina Association of Free and Charitable Clinics
NIMHD: the National Institute on Minority Health and Health Disparities
NQF: National Quality Forum
Roadmap: Quality of Care in Free and Charitable Clinics: Roadmap to Health Equity
SD: standard deviation
UDS: Uniform Data System
